# A Rare Case of Anterior Semicircular Canal BPPV Resistant to Treatment: A Case Report and Literature Review

**DOI:** 10.3390/audiolres15050126

**Published:** 2025-09-28

**Authors:** Juras Jocys, Aistė Paškonienė, Eugenijus Lesinskas

**Affiliations:** 1 Faculty of Medicine, Vilnius University, LT-03101 Vilnius, Lithuania; 2 Clinic of Ear, Nose, Throat, and Eye Diseases, Institute of Clinical Medicine, Faculty of Medicine, Vilnius University, Santariskiu 2, LT-08661 Vilnius, Lithuania

**Keywords:** anterior semicircular canal, benign paroxysmal positional vertigo, ASC-BPPV, apogeotropic PSC-BPPV, posterior semicircular canal, refractory vertigo, refractory BPPV, down-beating nystagmus, torsional nystagmus, central positional nystagmus, particle repositioning maneuvers, mechanical rotational chair, vestibular disorders

## Abstract

**Background and Clinical Significance:** Benign paroxysmal positional vertigo (BPPV) most commonly involves the posterior semicircular canal (PSC), whereas anterior semicircular canal BPPV (ASC-BPPV) is rare, accounting for only 1–3% of cases. Most ASC-BPPV cases respond well to particle repositioning maneuvers (PRMs), with refractory presentations being exceptional and diagnostically challenging, particularly when differential diagnoses such as apogeotropic posterior semicircular canal BPPV (PSC-BPPV) or central causes must be excluded. **Case Presentation:** A 43-year-old woman presented with vertigo triggered by head extension and rolling in bed. Initial neurological and otoneurological examinations were unremarkable. During the left Dix–Hallpike maneuver, a vertical down-beating nystagmus with subtle leftward torsion appeared after a 5 s latency and lasted 15 s. The supine head-hanging maneuver provoked a stronger and longer 30 s response, while the right Dix–Hallpike was negative. Despite repeated PRMs, including Yacovino (Deep Head-hanging), reverse Epley, Epley, and modified Semont maneuvers, the patient remained symptomatic over three years. Intermittently, conversion to PSC-BPPV was suspected, and temporary resolution was achieved after left-sided Epley and Semont maneuvers, but recurrence followed. Treatment with a mechanical rotational chair (TRV) initially resolved symptoms, but vertigo recurred several months later following two syncopal episodes with minor trauma. Extensive neurological evaluation, including MRI, CT, EEG, and vascular ultrasound, excluded central causes. **Conclusions:** This case illustrates the diagnostic and therapeutic difficulties posed by refractory ASC-BPPV, particularly in differentiating it from apogeotropic PSC-BPPV and central etiologies. It underscores the importance of latency, torsional characteristics, and supine head-hanging testing in diagnosis and demonstrates the potential role of mechanical rotational chairs in management. Personalized approaches incorporating anatomical imaging and maneuver adaptation are essential in such complex cases.

## 1. Introduction

Benign paroxysmal positional vertigo (BPPV) is the most common cause of peripheral vertigo that accounts for almost half of the cases, with approximately 80 to 90% of cases associated with canalolithiasis of the posterior semicircular canal (PSC) [1]. Lateral semicircular canal BPPV (LSC-BPPV) and anterior semicircular canal BPPV (ASC-BPPV) are less common variants accounting for an estimated 10 to 20% and 1 to 2% of all BPPV cases, respectively [1]. The rarity of ASC-BPPV is thought to be due to the anatomical orientation of the anterior semicircular canal, which typically prevents otolith debris from entering the canal during most daily activities. While the clinical characteristics and treatment options of PSC and LSC-BPPV are well established due to their higher prevalence, the literature on ASC-BPPV remains limited, with predominantly small case series reporting that most cases are usually easily resolved with manual particle repositioning maneuvers. Apogeotropic PSC-BPPV may also present with down-beating nystagmus and contralateral torsion, mimicking ASC-BPPV and further complicating the diagnostic process [2]. This case report contributes to the limited body of literature by presenting an unusual case of ASC-BPPV resistant to intensive conventional manual repositioning maneuvers performed over three years. To our knowledge, reports of ASC-BPPV cases with this degree of treatment refractoriness are rare.

## 2. Case Presentation

A 43-year-old healthy Caucasian woman with no chronic medical conditions and no regular medication use presented with vertigo triggered by head movements, particularly during head extension. The patient reported that symptoms often occurred at night, when rolling over in bed or lying down. Neurological examination and computed tomography (CT) imaging of the brain were unremarkable. Otoneurological examination revealed clear external auditory canals bilaterally, tympanic membranes appearing gray and exhibiting a normal light reflex. Vestibulospinal (VSR) reflexes were stable. The head-shaking test (HST) showed no abnormalities. The head impulse test (HIT), along with the video head impulse test (vHIT), was normal (horizontal canal gain at 60 ms AD/AS > 0.75), indicating an intact vestibulo-ocular reflex (VOR). Videonystagmography (VNG) revealed no spontaneous nystagmus (SNy), the caloric test was normal (UW/DP < 25%), and the oculomotor test was normal. Tympanogram types AD/AS were A, and pure-tone audiometry results were within the normal limits.

Given the clinical suspicion of BPPV, a series of provocative maneuvers was performed. During the left-sided Dix–Hallpike maneuver, after a latency period of approximately 5 s, the patient experienced nausea and vertigo accompanied by a vertical down-beating nystagmus with a subtle leftward torsional component (from the patient’s point of view), lasting around 15 s (Table 1). The right-sided Dix–Hallpike maneuver was subsequently performed—it did not provoke vertigo or nystagmus (Supplemental Video S1). These findings were suggestive of left ASC-BPPV and prompted further evaluation with the supine head-hanging maneuver. In this position, nystagmus again appeared after a latency of approximately 5 s but was longer in duration (around 30 s) and more easily discernible (Supplemental Videos S2 and S3). The duration of the episode further reaffirmed the possibility of ASC canalolithiasis. Given the presence of down-beating nystagmus with a leftward torional component, additional neurological investigations, including brain MRI, were necessary to exclude central nervous system pathology.

These findings made involvement of the right posterior semicircular canal (apogeotropic PSC-BPPV), which can mimic left anterior canal BPPV, less likely. Given the high probability of left ASC-BPPV, Yacovino (Deep-Head-Hanging) and reverse Epley maneuvers were carried out first. Despite repeated attempts, neither maneuver resulted in sustained symptom resolution or elimination of nystagmus during provocative testing. Because apogeotropic right PSC-BPPV could not be entirely excluded, a right-sided Epley maneuver, right reverse Epley, and a modified Semont maneuver (toward the affected side to move debris out of the non-ampullary arm toward the common crus/utricle) were therefore performed for this possibility. During the Yacovino maneuver, in the supine head-hanging position, a down-beating nystagmus with a subtle leftward torsional component was consistently observed and appeared more clearly than with the other maneuvers, without inversion upon returning to the upright sitting position. The patient’s symptoms persisted despite these repeated interventions, and positional testing with the left Dix–Hallpike and supine head-hanging maneuvers continued to elicit the characteristic down-beating nystagmus and vertigo. Intensive repositioning therapy continued over the following year, with the patient returning to the hospital on more than 15 occasions. Despite repeated attempts with the Epley, reverse Epley, Yacovino (Deep Head-Hanging), and modified Semont maneuvers, the condition persisted and remained refractory to treatment.

Because a central cause remained a major differential diagnosis, a more detailed neurological work-up was pursued. As noted earlier, neurological examination was unremarkable, without focal deficits. Brain CT revealed no sub- or supratentorial density changes, while both brain and temporal bone MRI were normal. In addition, extracranial vascular ultrasound showed normal arterial flow without abnormalities.

Repositioning therapy with the previously mentioned maneuvers was continued over the following two years, but clinical improvement remained minimal. Throughout these three years, the nystagmus was predominantly down-beating with a leftward torsional component. Intermittently, during the left-sided Dix–Hallpike maneuver, a transient episode of up-beating nystagmus with a more pronounced leftward torsional component was observed after a latency of approximately 5 s and lasting around 35 s (Table 1). This raised suspicion of conversion to left posterior semicircular canal BPPV, likely secondary to repeated and intensive repositioning maneuvers. A left-sided Epley maneuver and Semont maneuver were therefore performed, after which temporary resolution was achieved: neither the left Dix–Hallpike nor the supine head-hanging maneuvers elicited nystagmus or vertigo. Within several weeks, however, the patient again reported recurrence of symptoms, and at the subsequent hospital visit, provocative testing with the left Dix–Hallpike and supine head-hanging maneuvers once more elicited down-beating nystagmus with a subtle leftward torsional component.

After three and a half years of unsuccessful conservative treatment, the opportunity arose to perform repositioning maneuvers using a mechanical rotational chair (MRC). The patient underwent a series of maneuvers with the TRV chair: the Yacovino (Deep Head-Hanging) maneuver, the potentiated Epley maneuver, and the Semont maneuver. All maneuvers were performed according to the manufacturer’s recommendations. The deep head-hanging maneuver was carried out as described by Yacovino et al. and adapted to the TRV chair—the patient was rotated along the horizontal axis into a deep head-hanging position (supine, head extended 30–40° below horizontal plane), then elevated in 45° increments every 30 s [3,4]. The potentiated Epley maneuver consisted of ten sudden decelerations against the rubber stop of the TRV chair at each of the five 45° positions of a 180° turn before returning the patient to the upright position (Supplemental Video S4) [4]. The Semont maneuver was performed by rotating the chair along the horizontal axis to deliver a side-thrust movement, with the stop supports providing controlled deceleration, as described in the TRV chair technical documentation [5]. Repositioning therapy appeared successful following these interventions, as the patient no longer reported vertigo during routine head movements or during provocative testing. All of the TRV chair maneuvers (Yacovino, potentiated Epley, and Semont) were performed twice and during two treatment sessions over the course of two weeks, occurring three and a half years after the patient’s initial presentation.

After several months without symptoms, the patient experienced her first episode of syncope-like loss of consciousness. A brain CT scan revealed no focal density changes in the supra- or infratentorial regions and no signs of acute intracranial hemorrhage. Following the event, she recovered fully and did not experience a recurrence of BPPV symptoms, positional maneuvers (Dix-Hallpike, supine head-hanging) also failed to provoke vertigo. Given the negative imaging findings, normal neurologic examination, absence of focal neurological signs, and the absence of BPPV symptom recurrence, the syncopal episode was interpreted as likely vasovagal in origin [6].

Three months later, the patient experienced a recurrent syncopal episode while playing tennis that resulted in a minor head injury. The recurrence of syncope further cast doubt on the original diagnosis of ASC-BPPV and highlighted the need for a more thorough neurological evaluation to exclude central causes. The patient had not reported any symptoms previously associated with BPPV before the episode and remained alert, oriented, and able to follow all commands on examination. Pupils were equal and reactive to light, and ocular motility was unrestricted in all directions of gaze. Motor control of all limbs was normal, with good strength and no evidence of muscle atrophy. Deep tendon reflexes in the upper limbs (biceps, triceps, brachioradialis) and lower limbs (patellar and Achilles) were symmetric and present bilaterally. The patient was stable in the Romberg position and performed the finger-to-nose and heel-to-shin tests accurately. She reported no sensory disturbances.

A repeat brain CT scan showed no abnormalities, and laboratory testing was unremarkable. Previously performed brain MRI showed no structural abnormalities. No spontaneous nystagmus was present. EEG during wakefulness demonstrated predominantly 10–11 Hz alpha activity with occasional irregularities, and a single one-second paroxysm of slower, irregular, diffusely spreading waves with sharpened components was noted in the left central-parietal and right temporal regions. Photic stimulation and hyperventilation did not provoke additional abnormalities. Duplex ultrasound of the extracranial carotid and vertebral arteries confirmed patent common, internal, and external carotid arteries bilaterally, with no hemodynamically significant stenosis. Intima–media thickness was within normal limits, and vertebral artery flow was normal in direction bilaterally.

Following this event, vertigo symptoms reappeared during specific head movements, particularly with head extension. Provocative testing again elicited positional nystagmus, with it being most clearly discernible in the supine head-hanging position: after a latency of 4 s, a vertical down-beating nystagmus with a leftward torsional component lasting approximately 25 s.

With no central cause identified despite a thorough neurological evaluation, the clinical picture was most consistent with left ASC-BPPV. This conclusion was supported by the typical nystagmus pattern observed, which was most discernible and longest in duration in the supine head-hanging position and additionally by the intermittent conversion to posterior canal BPPV, as described in similar cases of ASC-BPPV, along with a slight torsional component compared to the torsional component typically present with apogeotropic PSC-BPPV. A neurological cause for the syncope was not identified, and the most likely diagnosis remains vasovagal syncope. The patient is currently still experiencing symptoms following the recurrence of left ASC-BPPV—an intensive otolith repositioning therapy program is planned.

## 3. Discussion

BPPV is caused by the detachment of otoliths, which under normal conditions remain attached to the maculae of the utricle and saccule. Due to its anatomical orientation, the posterior semicircular canal is the most common site of otolith accumulation, followed by the horizontal canal, while the anterior semicircular canal is the least commonly affected. The free-floating otoliths disrupt normal endolymphatic flow during head movements, leading to recurrent, brief episodes of vertigo triggered by actions such as lying down, looking up, or turning over in bed [1,2]. Once otoconial debris enters the canal, it may move freely within either the short arm or the long arm, producing canalithiasis, or it may adhere to the cupula, resulting in cupulolithiasis [2]. The distinction between these two types is based on the duration and latency of nystagmus: episodes lasting > 60 s with little or no latency are more typical of cupulolithiasis, whereas episodes lasting < 60 s with a clear latency period are characteristic of canalolithiasis [2]. The direction of the flow of the endolymph relative to the kinocilium determines either an excitatory or an inhibitory response that is conducted by the vestibular nerve [2].

ASC-BPPV is considered the rarest form of the condition, primarily due to the anatomical orientation of the anterior semicircular canal, which makes it difficult for otolith debris to enter and remain within the canal. The anterior semicircular canal is oriented nearly vertically when the person is in the upright position, which allows debris to exit more easily. Rare cases of ASC-BPPV are thought to result from individual anatomical variations in the semicircular canals that may predispose certain individuals to otolith accumulation in the anterior canal [1]. Compared to the posterior and horizontal canal variants, data on the prevalence, diagnostic methods, and therapeutic maneuvers for ASC-BPPV are limited, with many studies including only small numbers of patients. Cases in which ASC-BPPV remains refractory to manual PRM treatment are even more exceptional.

Diagnosis of ASC-BPPV is made possible by employing provocative Dix–Hallpike or supine head-hanging maneuvers, during which the head is extended [2,7,8]. This variant typically presents with vertical down-beating nystagmus that is often accompanied by a subtle torsional component that may assist in identifying the affected side [2,7,8,9,10,11,12]. However, the torsional component is frequently very subtle, which often makes it difficult to ascertain its direction. A rightward torsional motion (from the patient’s perspective) suggests involvement of the right anterior canal, whereas a leftward torsional motion indicates left-sided involvement [2,8].

The diagnosis of ASC-BPPV relies on the presence of a characteristic nystagmus pattern together with the exclusion of alternative causes that may mimic it. In this case, where the presumed BPPV was refractory to conservative treatment, the primary differential diagnoses included central nervous system pathologies involving the brainstem or cerebellum, as well as inhibition of the contralateral posterior semicircular canal (apogeotropic PSC-BPPV), which can reproduce a nystagmus pattern similar to that of ASC-BPPV [2,8,13].

**Figure 1:**
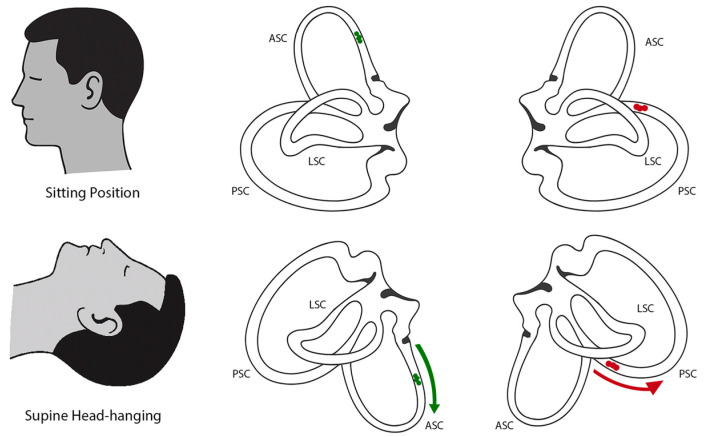
Schematic illustration of semicircular canal orientation and otolith movement in different positions. (**Top row**) Bilateral projections of the semicircular canals in the upright sitting position. (**Bottom row**) Bilateral projections in the supine head-hanging position. In the left anterior canal, otoconia (green) movement is ampullofugal (green arrow), producing excitatory endolymph flow and resulting in down-beating nystagmus with a leftward torsional component (consistent with ASC-BPPV). In the right posterior canal, otoconia (red) located in the non-ampullary arm move ampullopetally (red arrow) toward the ampulla, producing inhibitory flow (apogeotropic PSC-BPPV), which can mimic the same clinical presentation of down-beating nystagmus with a leftward torsional component [10,14].

Inhibition of the contralateral posterior canal (apogeotropic PSC-BPPV) may present with down-beating nystagmus and contralateral torsion during Dix–Hallpike testing and the supine head-hanging test, because in these positions the anterior canal and the contralateral posterior canal lie in the same plane—this overlap can mimic the nystagmus pattern typically attributed to ASC-BPPV (Figure 1) [2,8,11,14]. In apogeotropic PSC-BPPV, the typical nystagmus and symptoms may appear in one or both Dix–Hallpike positions and are occasionally provoked during the supine head-hanging test [2,8]. In ASC-BPPV, by contrast, the canal may be stimulated in both Dix–Hallpike positions, but responses are often weaker or absent on one side. In contrast, the supine head-hanging position consistently provokes the characteristic nystagmus and is considered the most sensitive test [2,8]. Another useful distinction is the torsional component, which tends to be more pronounced in apogeotropic PSC-BPPV than in ASC-BPPV [2]. Canalithiasis of the anterior canal usually demonstrates a short but distinct latency period of approximately 2–5 s, whereas in apogeotropic PSC the onset of nystagmus can more often be immediate or with minimal latency [8,11,15,16]. In our case, although the Dix–Hallpike test was positive only on the left, the torsional component was subtle, and the strongest and longest-lasting nystagmus was observed in the supine head-hanging test, with a latency of around 5 s, no inversion of nystagmus upon returning to the sitting position—findings that pointed more toward ASC-BPPV [2,8,11].

Central nervous system causes of positional nystagmus, most often related to cerebellar or brainstem lesions, were essential to exclude—even more so after the syncopal episodes. Features such as immediate onset without latency, persistence without fatigability, and the absence of a consistent torsional component are more suggestive of a central etiology [12,13]. In comparison, our patient consistently demonstrated a latency of several seconds, limited duration, fatigability, and a reproducible torsional component, which favored a peripheral origin. The absence of focal neurological signs on repeated examinations, together with normal findings on CT, MRI, EEG, and vascular ultrasound, further reduced the likelihood of a central cause. Vestibular evoked myogenic potentials (VEMPs) were not performed as the test was not available, they may provide additional information given the contribution of otolith pathways to vestibular-mediated autonomic responses and vasovagal syncope [6].

Treatment options for ASC-BPPV involve PRMs aimed at redirecting displaced otoliths back into the utricle or saccule. The maneuvers most commonly described in the literature for treating ASC-BPPV include the Yacovino (Deep Head-Hanging) maneuver, the Epley maneuver, and the reverse Epley maneuver [1,17]. However, in complex or refractory cases where standard maneuvers prove ineffective, clinicians often apply modified versions of these techniques in an effort to achieve resolution of the condition. Compared to PSC-BPPV, for which treatment efficacy and success rates have been thoroughly studied, data on ASC-BPPV remain limited, mainly because of the rarity of this variant and the scarcity of large-scale studies. Nevertheless, a systematic review utilizing descriptive analysis reported a success rate exceeding 75% for the aforementioned maneuvers in the treatment of ASC-BPPV [1].

Because apogeotropic PSC-BPPV remained a diagnostic possibility, therapeutic maneuvers specific to this variant were also attempted. Conventional maneuvers for typical PSC-BPPV, such as the standard Epley and Semont, are usually ineffective in the apogeotropic form, as otoconia are usually located in the non-ampullary arm of the canal [8,15,16]. A right-sided reverse Epley maneuver and a modified Semont maneuver were performed in our patient. These maneuvers are designed to promote debris migration from the non-ampullary arm toward the common crus and ultimately the utricle, either resolving the condition directly or converting it into typical PSC-BPPV, which can then be treated with conventional maneuvers [8,15].

Even with the high overall success rate of PRMs for the treatment of BPPV, an estimated 12.5% of patients remain non-responsive to treatment and continue to experience lingering symptoms [7]. As demonstrated in our case, this can lead to repeated clinical visits, prolonged periods of impaired quality of life, and a significant psychological burden for the patient. Personalized medicine approaches that are tailored to the individual anatomical variations in the semicircular canals represent the future of treatment for these refractory cases [7].

Mechanical rotational chairs (MRCs) have emerged as a novel option for the diagnosis and treatment of BPPV. Several studies have compared the effectiveness of MRCs with conventional PRMs [5,18,19]. The majority of the literature agrees that MRCs serve as a valuable tool, particularly in the diagnosis and management of BPPV in specific subpopulations or complex cases. However, in uncomplicated cases, they do not significantly outperform manual maneuvers [19]. A recent study investigating the use of the TRV chair for refractory BPPV found that patients in this group exhibited statistically significant anatomical variations in the angles of their semicircular canals [20]. The study concluded that the TRV chair was an effective therapeutic option in such cases—21.4% of patients achieved objective resolution, and 42.9% reported symptom relief following the intervention [20].

This case report further illustrates the utility of this modality in managing refractory BPPV when all conventional manual repositioning techniques have failed. MRCs enable precise and controlled head movements that can achieve angles not possible through manual maneuvers, resulting in a significant therapeutic advantage in anatomically challenging cases. These observations suggest that the effectiveness of interventions using the MRCs relies not only on the technology itself but also on the clinician’s skill and the availability of advanced MRI or CT imaging protocols in order to assess the precise anatomy of the semicircular canals. Such imaging allows clinicians to tailor repositioning maneuvers to the individual anatomical variations in each patient, which in turn increases the likelihood of successful treatment.

## 4. Conclusions

This case report contributes to the limited literature on the rare presentation of treatment-refractory ASC-BPPV. The need to carefully differentiate ASC-BPPV from central nervous system pathology and apogeotropic PSC-BPPV illustrates the complexity of the diagnostic process. The case also demonstrates that mechanical rotational chairs can be a valuable tool in the management of complex or refractory cases when conventional maneuvers have failed. Additionally, it further supports that ASC-BPPV typically presents with vertical down-beating nystagmus, which typically should be accompanied by a subtle torsional component with a latency period lasting 2–5 s. The persistence and refractoriness observed here may be attributable to anatomical variations in the semicircular canals, highlighting the importance of personalized approaches that integrate advanced imaging, individualized maneuver modifications, and clinician expertise. Continued reporting of similar cases and case series, together with the development of advanced imaging protocols to better visualize semicircular canal anatomy, are essential for refining treatment, differential diagnosis strategies and improving patient outcomes in ASC-BPPV as well as other refractory forms and rare forms of BPPV.

## Figures and Tables

**Table 1:** Provocative maneuver responses.

Maneuver	Latency	Duration	Nystagmus	Notes
Left Dix-Hallpike	~5 s	~15 s	Down-beating + left torsion	Provoked vertigo and nausea
Right Dix-Hallpike	-	-	-	Did not provoke symptoms or nystagmus
Supine Head-hanging	~5 s	~30 s	Stronger down-beating + left torsion	No inversion on returning upright, longest, most discernible
Left Dix-Hallpike	~5 s	~35 s	Up-beating + left torsion	Intermittent conversion to left PSC-BPPV

## Data Availability

The original contributions presented in this study are included in the article. Further inquiries can be directed to the corresponding author.

## Supplementary Materials

The following supporting information can be downloaded at: https://www.mdpi.com/article/10.3390/audiolres15050126/s1.
