# Supplementary material for: A Rare Case of Anterior Semicircular Canal BPPV Resistant to Treatment: A Case Report and Literature Review
Source: Audiol Res. 2025 Sep 28;15(5):126. doi: 10.3390/audiolres15050126 (PMC12561029; doi:10.3390/audiolres15050126)
Supplement: Supplementary file 1 [file audiolres-15-00126-s001.zip › Supplemental_materials/Legend.pdf]

Supplemental videos (Legend)

Video S1. Right Dix-Hallpike maneuver eliciting no nystagmus or vertigo.

Video S2. Yacovino (Deep-Head-hanging) maneuver eliciting vertical down-beating nystagmus with a subtle leftward torsional component in the supine head-hanging position.

Video S3. Close up version of vertical down-beating nystagmus with a subtle leftward torsional component in the supine head-hanging position.

Video S4. Potentiated Epley particle repositioning maneuver performed on the patient using a mechanical rotational TRV chair.
