# Supplementary figures and images for: A Rare Case of Anterior Semicircular Canal BPPV Resistant to Treatment: A Case Report and Literature Review
Source: Audiol Res. 2025 Sep 28;15(5):126. doi: 10.3390/audiolres15050126 (PMC12561029; doi:10.3390/audiolres15050126)

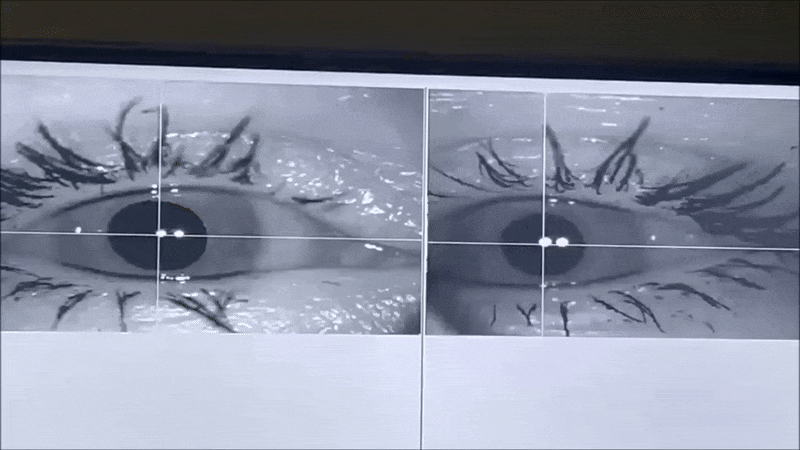

Supplement: Supplementary file 1 [file audiolres-15-00126-s001.zip › Supplemental_materials/Supplementary_video_3.gif]
